# Assessment and Implementation of WOAH Day 1 Competencies (AID-1C): a cyclical methodology for curriculum harmonization with international standards

**DOI:** 10.3389/fvets.2024.1390779

**Published:** 2024-05-31

**Authors:** Armando E. Hoet, Samantha Swisher, Amanda M. Berrian, Andrea L. Bessler, Ivana Grozdic

**Affiliations:** ^1^Veterinary Public Health Program, Department of Veterinary Preventive Medicine, College of Veterinary Medicine, The Ohio State University, Columbus, OH, United States; ^2^Division of Epidemiology, College of Public Health, The Ohio State University, Columbus, OH, United States

**Keywords:** curriculum evaluation, curriculum revision, competency assessment, WOAH day 1 competencies, veterinary public health, veterinary preventive medicine

## Abstract

**Introduction:**

The World Organisation for Animal Health (WOAH) Day 1 Competencies for Graduating Veterinarians provide a standard framework to guide Veterinary Educational Establishments (VEEs) in improving their veterinary public health and population medicine curricula. However, pursuing a curriculum revision to incorporate these standards may be daunting, especially for institutions with limited resources or experience. This manuscript describes a methodology for targeted curriculum revision specifically focused on the WOAH Day 1 Competencies.

**Phases of the AID-1 process:**

The Assessment and Implementation of WOAH Day 1 Competencies (AID-1C) is a six-step, cyclical, collaborative methodology that encompasses a series of tools and processes that help a VEE to evaluate their curriculum, identify and prioritize gaps, and develop and implement an action plan based on the results. The six phases of the AID-1C process include: (1) Assessment of the proficiency of the VEE’s graduates in Day 1 Competencies using a structured Evaluation Tool; (2) A systematic curricular review and evaluation; (3) Identification and prioritization of interventions through a group problem-solving and prioritization exercise called Focus Forward; (4) Design and development of interventions to address identified gaps; (5) Curricular implementation; and (6) Monitoring and evaluation. The AID-1C methodology relies upon active involvement of senior students, recent graduates, faculty, instructional staff, and employers throughout the process.

**Conclusion:**

The AID-1C methodology provides a systematic, participatory, collaborative approach that simplifies the planning and execution of the curricular revision, making a complex process more manageable. This enables VEEs to improve their curricula, while moving toward harmonization with WOAH standards. The result is a curriculum that allows a VEE to train well-rounded and competent veterinarians, with the requisite skills to support the veterinary services in their country.

## Introduction

Curriculum assessment and revision are critical activities that help veterinary educational establishments (VEEs)^1^ ensure that they are graduating veterinarians who are well-equipped to meet current and future animal health challenges. There are many reasons that a VEE might choose to undertake curricular revision, including meeting institutional mandates, accommodating shifting administrative or professional priorities, addressing curricular drift, incorporating new pedagogical best practices, and assuring that materials reflect the latest scientific advances (1–4). Curriculum revision can take many forms, from large scale changes affecting the whole curriculum to more targeted interventions that are focused on a particular subject area.

This manuscript describes a methodology for targeted curriculum revision specifically focused on veterinary public health and population medicine. In a globalized society where infectious diseases can travel around the world in a matter of hours, having a veterinary workforce that is competent in these focus areas is critical to protect global health security (5–9). To support countries in improving the capacity of their veterinary workforce, the World Organization for Animal Health (WOAH) has outlined a series of non-clinical “Day 1 Competencies” (10) to prepare new veterinary graduates to effectively support veterinary regulatory and population health activities in their countries. These recommendations are also supported by a model curriculum that outlines key topic areas and when they should be taught in the curriculum (11).

The creation of these guidance documents in 2012–2013 represented an important step forward because they provide a clearly defined international standard for veterinary public health education. However, many VEEs needed to undertake significant curricular revisions to meet them, and this was a daunting task for faculty and administrators, especially those at institutions with limited resources or experience with curricular revision. Several examples exist of institutions conducting curriculum mapping to reach a preliminary assessment of how well their curriculum was aligned with the Day 1 Competencies (12, 13). However, using curriculum mapping as the sole method of evaluation, as these publications did, does not allow the VEE to assess the level of proficiency of their graduates, nor does it provide a mechanism for VEEs to identify and prioritize interventions to address gaps.

Therefore, there was a need for a standardized process that would allow VEEs to assess the competence of their current graduates and create a customized roadmap for moving toward compliance with the new standards. Because each individual VEE faces unique challenges, this process needed to be customizable and allow input from a wide range of local partners. It was also important that harmonization with the Day 1 Competencies be treated as an ongoing process rather than an endpoint, allowing VEEs to progress toward harmonization at a pace that was feasible for them and to adapt their plans in the future as the standards evolve.

To meet these needs, The Ohio State University College of Veterinary Medicine developed the *Assessment and Implementation of WOAH Day 1 Competencies* (AID-1C) methodology, a cyclical, collaborative methodology to help VEEs evaluate their curricula and move toward harmonization with WOAH standards using a systematic approach. The AID-1C methodology encompasses a series of tools and processes that help a VEE to evaluate their curriculum, identify and prioritize gaps, and develop an action plan based on the results. The methodology was originally developed for use at the University of Gondar in Ethiopia (14, 15), but it has recently been successfully applied in Southeast Asia, where it has been endorsed by the ASEAN Veterinary Statutory Body Network as a mechanism to implement a minimum accreditation standard in the region. Individual components of the methodology have also been adapted to help VEEs in South America and Central Asia to align their antimicrobial resistance curricula with the AAVMC/APLU AMR Learning Outcomes (16), demonstrating its flexibility and applicability in a variety of settings.

## Conceptualization of the AID-1C methodology

In 2015, the University of Gondar and The Ohio State University were selected by WOAH to participate in the first Veterinary Education Twinning Program in Africa (14). In order to remain accountable to donors and other stakeholders, it was important to be able to describe and visualize the process that would be used to assess and improve the veterinary training program at University of Gondar. The AID-1C methodology was developed to formalize a process that evolved organically during the Twining Program. The details of how this process unfolded are described in the series of technical reports associated with the UoG-OSU Twinning program (17–21).

**Figure 1:**
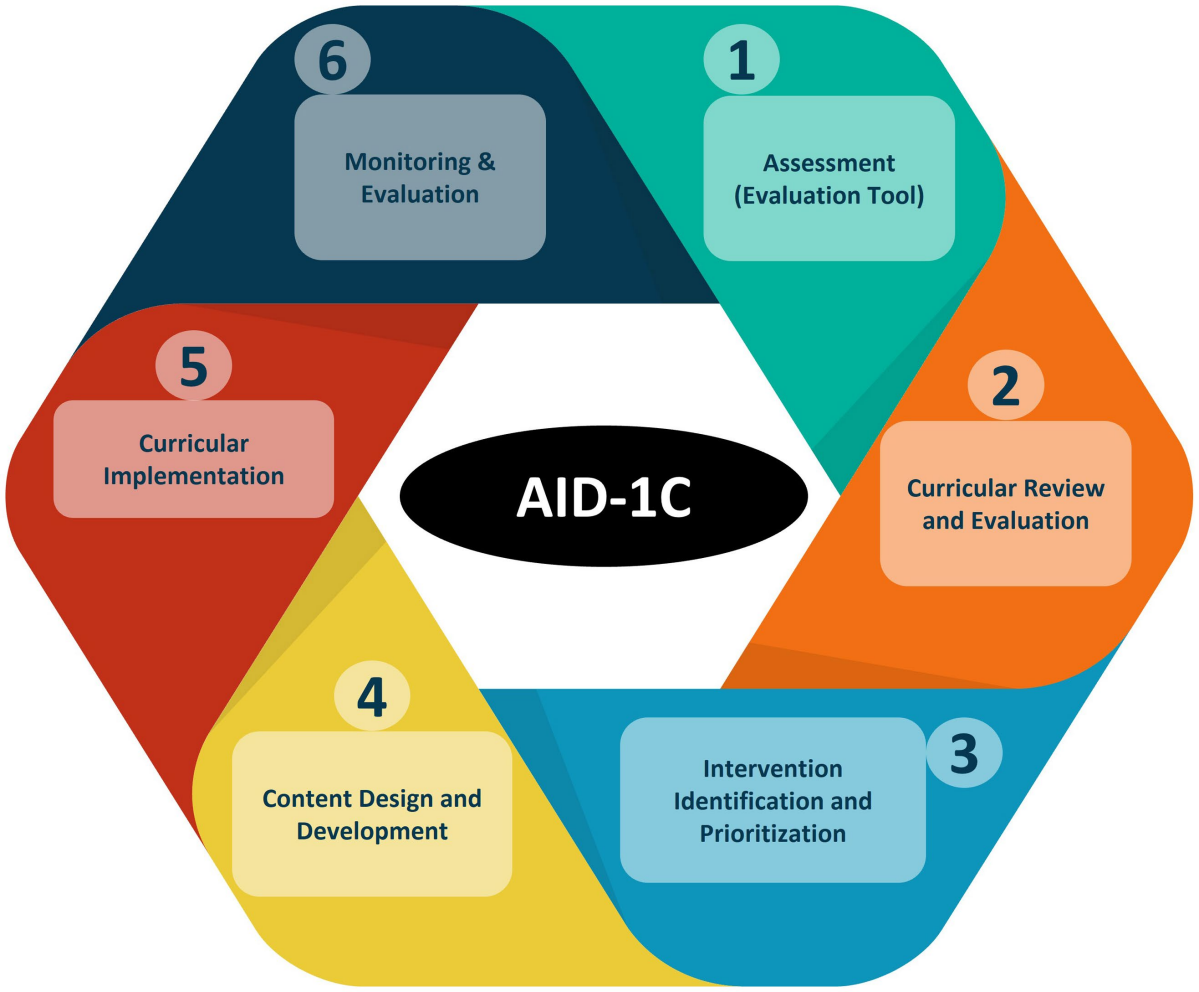
Cycle of the assessment and implementation of Day 1 competencies (AID-1C) methodology depicting the 6 stages of curriculum revision.

This manuscript describes the six phases of the AID-1C methodology (Figure 1), with examples of how they have been applied and adapted in different settings.

## Phases of the AID-1C Methodology

Before beginning the AID-1C process, it is necessary to assemble the *Curriculum Revision Team* that will shepherd the VEE through the curriculum revision (15). This team should include faculty, instructors, and staff who are involved in the teaching of WOAH Day 1 Competencies throughout the curriculum. This group should be interdisciplinary and diverse, reflecting different generations, ranks, and genders, as well as any other characteristics that are relevant for that institution. Careful selection of these members is critical to help reduce bias, incorporate different perspectives, and generate innovative ideas, among many other benefits.

### Phase 1—Assessment (Evaluation Tool)

The first phase of the AID-1C methodology is an evaluation of the proficiency of the VEE’s graduates in the areas outlined in the Day 1 Competencies. Best practices in veterinary education indicate that the most effective way to assess student competence is direct assessment through structured examinations, in which the students demonstrate their ability to successfully execute the relevant skills (22–24). However, the Day 1 Competencies comprise a wide variety of topics, many of which are abstract and not conducive to practical demonstration (e.g., Competency 3.1: “Organization of Veterinary Services – having a general awareness of and appreciation for the delivery of National Veterinary Services as a global public good”) (10). Therefore, evaluating all Day 1 Competencies through direct assessment of students is not always feasible, especially for VEEs in low- resource settings and those that have limited experience with this method of assessment.

Therefore, the AID-1C methodology relies upon senior students, recent graduates, faculty, and employers of the graduates to report on the perceived competence of the average new graduate based on their own experiences. Assessors are guided through a standardized, digital or paper-based survey [“The Evaluation Tool for WOAH Day 1 Graduating Veterinarian Competencies” (15)] in a facilitated Assessment Workshop. This Evaluation Tool consists of 175 questions that systematically evaluate the average new graduate’s knowledge and ability to perform a variety of skills, ranging from epidemiological calculations to carcass inspection to risk communication.

For each question, the assessor can indicate if a particular topic is not covered in the curriculum, or if they are unable to confidently evaluate the graduates on a particular topic. If they decide that the topic is indeed in the curriculum, then they are asked to rate the average new graduate on a five-point scale, ranging from “not competent” to “highly competent.” Competency levels are defined by the amount of assistance that a new graduate would require to complete the task on their first day after graduation.

Assessing competence indirectly in this manner reduces the resources required for the assessment, but it also means that assessor selection and facilitator training are essential to minimize bias. The inclusion of a diverse group of assessors representing students, recent graduates, faculty/instructors, and employers (from both the public and private sectors) helps to provide balance in the responses. Furthermore, careful evaluation of topics that have discordant results between these groups of assessors can yield important insights about potential areas for improvement. For example, if employers rate the graduates as proficient communicators, while the students self-report that they are not proficient, this discrepancy suggests the need for additional activities in the curriculum to build confidence in their communication skills. Having trained facilitators on hand during the workshop allows for the provision of additional examples or clarification as needed, which helps to improve the quality and validity of the data. The Evaluation Tool also includes open text fields for respondents to provide additional context to clarify their responses. The results of the assessment are always interpreted in conjunction with curriculum review (AID-1C Phase 2).

### Phase 2—Curricular review and evaluation

A full review of the official curriculum provides important context to interpret the results of the competency assessment conducted in Phase 1. The curriculum review begins with curriculum mapping, in which the *Curriculum Revision Team* reviews the curriculum and course syllabi to determine if, when, and in what depth topics relevant to the Day 1 Competencies are covered. The timing of delivery is also evaluated for alignment with the WOAH Curriculum.

**Figure 2:**
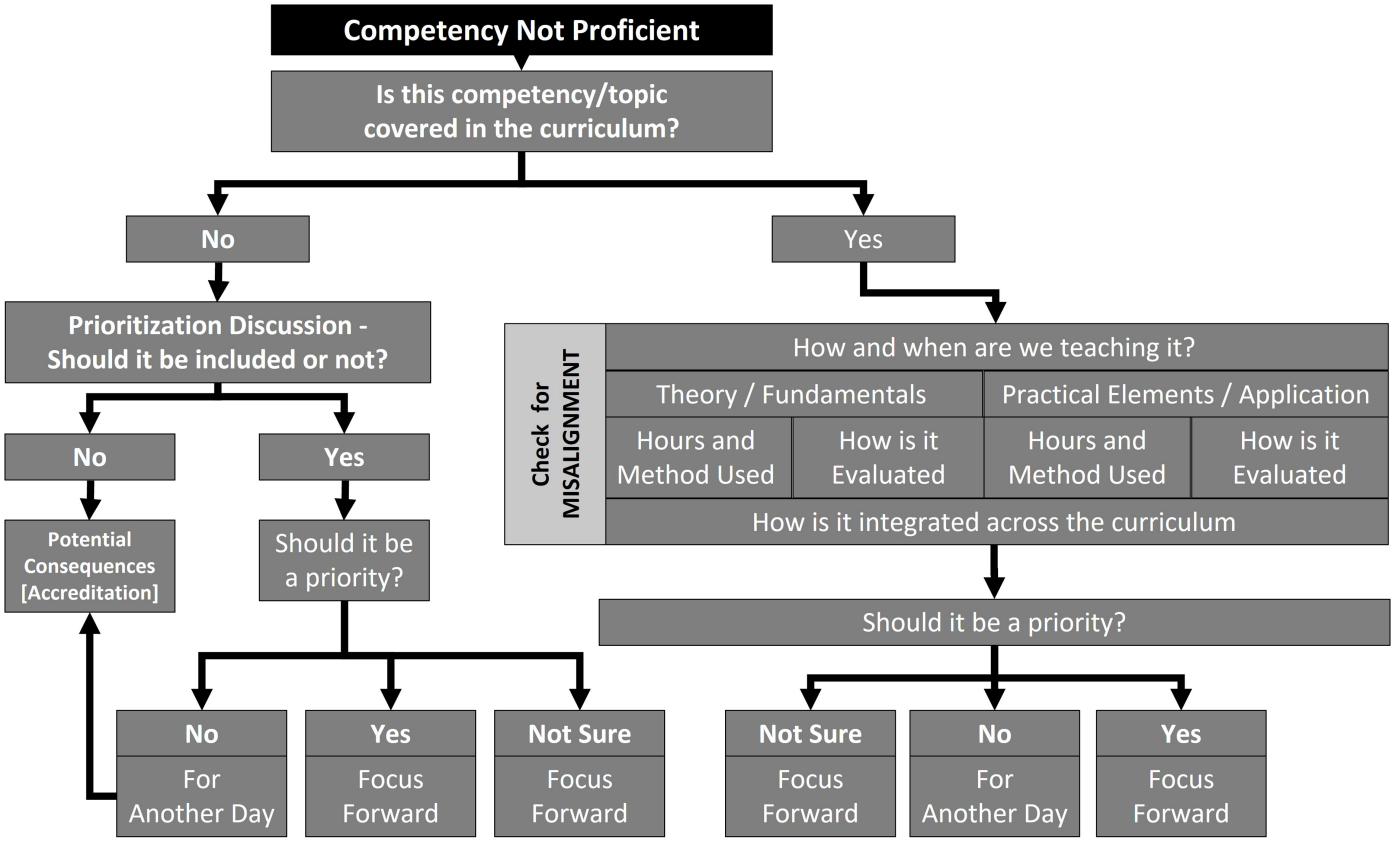
Algorithm for integrated interpretation of results obtained during Phase 1 (new veterinary graduate competency evaluation) and Phase 2 (curricular mapping) of the assessment and implementation of Day 1 competencies (AID-1C) methodology.

The team then compiles a list of topics that performed poorly in Phase 1, either because respondents *indicated either that* recent graduates were inadequately competent or that the topic was not taught in the curriculum. Each topic is cross-referenced with the curriculum map to determine if and when the topic is taught, what pedagogical and assessment methods are used, and whether the topic is vertically integrated through the different years of the curriculum (see Figure 2). As part of this analysis, particular attention is paid to the amount and type of applied training associated with the different topics. If the team agrees that coverage of the topic in the curriculum is insufficient, they perform a preliminary assessment of how important the gap is to address; for instance, in some countries only government veterinarians write health certificates and they receive on-the-job training for this task, so incorporating this topic into the curriculum may not be considered a high priority.

While the AID-1C methodology is primarily focused on identifying gaps and areas for improvement, it is also important to dedicate time during this phase to note the topics that performed particularly well. Understanding the factors contributing to the success of these topics can inform the development of strategies to approach the teaching of those that did not perform as well. Clearly identifying which topics do not require further intervention also helps to reduce unnecessary expenditure of time and resources updating curriculum materials that already meet needs and conform with international standards.

### Phase 3—Intervention identification and prioritization (Focus Forward)

Once the *Curriculum Revision Team* has identified key areas of concern based on integrating the results of Phases 1 and 2, internal and external partners are invited to participate in a two-day group problem-solving and prioritization exercise called Focus Forward. The ultimate goal of this exercise is to develop an action plan to improve the curriculum. This is achieved through five steps: (1) Socialization of assessment results, (2) Root cause determination, (3) Intervention identification, (4) Prioritization, and (5) Action Plan development.

Before the event, the coordinators organize the results of Phases 1–2 for presentation in themed sessions during the first day of the Focus Forward. A moderator introduces the gaps for each session (Step 1: Socialization of assessment results), then encourages participants to engage in small group discussions, in which they consider the reasons for the gaps and potential solutions to address them. Each group consists of 6 to 8 participants representing faculty, recent graduates, government officials, private sector representatives, and other boundary partners. Having these different perspectives helps to ensure that the groups can think creatively and identify innovative solutions, while also remaining rooted in a practical understanding of the academic program.

This process is facilitated by providing the participants with specially designed questions to guide the discussion. The initial questions focus on discussing the underlying causes and potential barriers associated with these problems (Step 2: Root cause determination). Each one of these questions is paired with a follow up prompt for participants to identify potential solutions and interventions (Step 3: Intervention identification) that address the causes identified by the participants in the previous step. Participants are also encouraged to identify the institutions or individuals who would be the key partners in implementing these interventions. If the moderators have experience addressing similar gaps in other institutions, they may give examples of possible solutions during the introduction of this section, but the emphasis is on allowing the participants to identify solutions that are appropriate to their local context. The moderators also encourage the participants to propose solutions that are innovative but also feasible in the context of the VEE’s needs and capabilities. These three steps take place during the first day of the Focus Forward workshop.

Following the activities on the first day, the event coordinators collect all the suggested ideas and organize them for the second day. During the second day, the moderator presents these ideas to the participants, who then use an anonymous real-time polling software to select the solution(s) that they feel should be prioritized (Step 4: Prioritization). The moderator instructs the participants to consider both the potential effectiveness of the intervention(s) and the feasibility (or likelihood of implementation) based on their current context or reality. After the event, the *Curriculum Revision Team* organizes the prioritized ideas and develops a short-term Action Plan (Step 5). For each identified gap, the Action Plan includes a detailed description of the corresponding intervention(s), including implementation strategy, timeline, and role assignments. This Action Plan is then shared with all participants and partners, including government agencies (e.g., Ministry of Education), other VEEs in the country, and the veterinary medical association(s).

### Phase 4—Design and development

During this phase, the interventions from the Action Plan are developed and expanded upon. For example, new teaching materials are created in preparation for introducing or expanding coverage of Day 1 Competency related topics. In some cases, this can be as straightforward as developing a few new lectures, but VEEs are encouraged to view this as an opportunity to incorporate pedagogical best-practices. This should include innovative and engaging teaching methods that allow students to learn by doing or by teaching others. These novel approaches are especially critical for topics that performed poorly in the assessment despite already being present in the curriculum. This is because opportunities to apply knowledge through problem-based learning or field exercises allow students to build the confidence that they need to be successful in these areas after graduation.

For VEEs in low-resource settings or with less experience in performing curricular revision, this phase can be an excellent opportunity for collaboration with more experienced VEEs. Such institutions often have access to more resources and may have experience and/or expertise in addressing similar gaps. However, such relationships will only be successful if there is true collaboration; input from the faculty or administrators who will be implementing the interventions is critical to assure that they are relevant and feasible to implement. The more experienced VEEs can also provide support in the form of continuing education for faculty covering (1) foundational information about new subject matter, (2) design and development of teaching materials, and/or (3) new pedagogical best practices to improve their teaching and students’ motivation.

### Phase 5—Curricular implementation

During this phase, the *Curriculum Revision Team* carefully considers the practical details of how the recommended changes will be implemented. For example, they determine where in the curriculum new material will be incorporated, who will be responsible for teaching it, and what material will be eliminated to accommodate it, if necessary. They also plan how they will transition from the old curriculum to the revised version and identify resources needed to support the transition. The level of planning required varies substantially depending on how extensive the revisions are; when a curriculum undergoes major restructuring, it can be quite challenging to manage overlapping needs for resources (professors with a particular expertise, teaching spaces, etc.) between different cohorts. When new materials and/or methods are introduced, instructors may require more support and time to prepare for classes, which can put a strain on low-resource VEEs where instructional staff are already stretched thin.

The duration of this implementation period will vary depending on the institutional or national processes required to review and approve amendments to the curriculum. The level of approval required often depends on the extent of the curricular changes proposed; if the changes represent a small proportion of the curriculum and/or mostly affect individual courses, then the approval will likely take place at the institutional level, which typically takes one to two years. On the other hand, if the changes are substantial (e.g., moving a core course to a different year), then a government agency and/or accrediting body may need to review and approve the changes before they can be implemented. In these situations, approvals can take several years, depending on the country’s legislative procedures.

Some changes can be implemented immediately, for example, modifying course content or implementing new teaching techniques. More extensive changes can only be implemented with a new cohort (e.g., moving a course to a different year, or changing core competencies) and therefore will take much longer to be fully implemented. As a result, this phase of the AID-1C methodology takes place on a much longer timescale than previous phases. It is usually necessary for multiple cohorts of veterinary students to progress through the program to allow the new curriculum to evolve and mature before the impact of the changes can be observed.

### Phase 6—Monitoring and evaluation

The main aim of the Monitoring & Evaluation process is to allow VEEs to identify and address issues in real time, rather than waiting four or more years for a full evaluation after a cohort has completed the revised curriculum in its entirety. Realtime feedback should be collected from students, faculty, and administrators after the first few iterations of a new course or activity under the new curriculum. Feedback should be collected using multiple mechanisms customized for each group, including anonymous surveys and focus groups. In the case of students, standard teaching evaluation surveys need to be modified to include specific questions assessing the new teaching methods and/or content that were introduced into the course. These evaluations should be supplemented by small focus groups to assess student morale, motivation, and satisfaction with the changes. Evaluations delivered to faculty aim to identify any obstacles and challenges encountered in the delivery of the new content and to assess the faculty’s perception of the value of the changes. Finally, administrators should be included in the evaluation process to determine the impact the new changes are having on the institution’s logistics, function, and budget.

### Restarting the cycle

After 2–3 cohorts have completed the new curriculum in its entirety, a full curriculum assessment should be repeated to determine how successfully the previously identified gaps or deficiencies have been addressed. Depending on the length of the VEE’s curriculum, this could be as many as 8–10 years later. During this assessment, Phases 1 and 2 are repeated as described above. It is important to note that this repeat assessment can be subject to response shift bias (25). For example, at the University of Gondar, many of the topics targeted for intervention performed worse during a follow-up assessment. However, when more qualitative feedback was elicited, it appeared that the declining quantitative results could be attributed to greater awareness by the participants of the importance of the Day 1 Competencies and the room that still existed for improvement.

## Example: Evaluation of student training in outbreak investigation

This section provides an example of the application of the AID-1C methodology to a specific topic, outbreak investigation, that represents a common challenge for many VEEs (including those in high-resource settings). Outbreak investigation is covered in the section of the Evaluation Tool pertaining to Epidemiology (WOAH Competency 2.1), but in reality, it incorporates subject matter from many different competencies, including Transboundary Animal Diseases (2.2), Zoonoses (including foodborne disease) (2.3), Emerging and Re-emerging Diseases (2.4), Food Hygiene (2.6), Management of Contagious Diseases (3.3), and Applications of Risk Analysis (3.5). The interdisciplinary and applied nature of this topic is one of the reasons that many VEEs struggle to teach it effectively, and the involvement of diverse partners in the AID-1C methodology is instrumental in assuring successful curriculum improvement.

Outbreak investigation is an example of a topic where there may be differences between groups in their assessment of new graduates during AID-1C Phase 1. Faculty may recall the extensive didactic instruction that they provide on this topic and indicate that new graduates are highly competent in outbreak investigation. Employers, by contrast, may feel that new graduates require substantial assistance and on-the-job training to apply this knowledge, and thus rate graduates as insufficiently or not competent. Because the Evaluation Tool stratifies results by participant group, such discrepancies are easily detected, and the subject can be flagged for more detailed review. Upon review of the curriculum in AID-1C Phase 2, it often becomes apparent that, although outbreak response is included in the curriculum, the coverage is primarily didactic and theoretical, with no opportunities for practical application. If curricular assessment were only based on curriculum mapping, without the additional perspective provided by the Assessment Workshop, it is very likely that this imbalance between theoretical and practical training would be missed.

If a lack of practical training in outbreak investigation is a gap that emerges from Phases 1 and 2, the issue is then presented to participants in the Focus Forward process (Phase 3), where internal and external partners work together to identify opportunities to deliver the material in a more dynamic and practical way. The involvement of external partners at this stage is important for two reasons: (1) their real-world experience provides valuable perspectives on what content should be included/emphasized and (2) their involvement at the inception of an intervention ensures that they are invested in the process and increases the likelihood that they will offer to assist with future stages.

During the design and development phase (Phase 4), many VEEs discover that their faculty may not have the expertise to design, deliver, and/or implement practical teaching methods, such as tabletop or field exercises, without external support. Common challenges include insufficient subject matter knowledge, limited real-life experience, and/or lack of expertise with hands-on teaching methods. Institutions that are less familiar with these methods of teaching often benefit from partnering with a more experienced VEE that can provide continuing education and mentorship to faculty as they develop new materials. Involving working professionals can also be very beneficial at this stage because the design of an effective and realistic tabletop or field exercise relies on input from individuals with lived experience. Collecting feedback from individuals who use these skills in their daily work, both in the public and private sectors, helps ensure that exercises are realistic and reflect current government or industry practice. These professionals can also help to identify priority issues for their organization or veterinary services; for example, suggesting a specific disease to be the focus of an outbreak exercise. In some cases, they may even be able to provide real scenarios that can serve as the basis for development of an exercise.

Implementation (Phase 5) of practical content to teach outbreak investigation can be challenging because hands on exercises typically require more resources than didactic teaching in the classroom. This includes physical materials (e.g., personal protective equipment, sampling supplies), but also access to appropriate facilities (e.g., farms, diagnostic labs) and qualified facilitators. The partnerships formed in earlier stages of the AID-1C process can be useful during this stage because working professionals often make good facilitators, and senior leaders from the public or private sector may be able to offer support in the form of access to facilities or supplies.

The AID-1C methodology’s emphasis on continuous monitoring and evaluation (Phase 6) means that VEEs have the opportunity to identify and address problems in real time. This is especially important in the implementation of practical and applied content because VEEs might not have significant prior experience with these methods. Therefore, it is essential to conduct evaluations immediately after each new intervention or activity to document students’ and faculty’s experiences and identify opportunities for improvement in real time. This active monitoring allows for continuous improvement as the changes in the new or updated curriculum are implemented. In addition to informing future iterations of the hands-on activities, monitoring student performance during practical exercises can help to identify opportunities to improve the coverage of related materials earlier in the curriculum. Because incorporating opportunities for practical application often makes student knowledge gaps more apparent, it is not uncommon for reports of perceived competency to temporarily decrease during this phase.

## Discussion and conclusions

The development of the WOAH Day 1 Competencies represented an important step forward because it provided a standard framework to guide VEEs in improving their veterinary public health curricula to support the veterinary services in their countries. Having an established process for collaboratively identifying, prioritizing, and addressing gaps can help galvanize institutions to begin the process of harmonizing their curriculum with WOAH standards.

The AID-1C methodology breaks down a very complex curricular revision and update process into manageable steps. Each phase has a discrete beginning and end, which helps the VEE community to stay motivated, as they feel that they are making progress. This methodology is supported by a suite of tools (e.g., Evaluation Tool), processes (e.g., Focus Forward), and defined deliverables (e.g., Action Plan) at each phase, which simplifies the planning and execution of the curricular revision, making a complex process more approachable.

Updating and improving the curriculum is a multiyear process that requires inputs from a large number of people. Therefore, it is essential to have a structured process that provides a roadmap with clear steps and outcomes. Because of the long period over which this process unfolds, there is likely to be leadership turnover; this is especially common in countries outside of the USA where the deans and administration often change every 4 to 6 years. Having a well-defined roadmap, as described in the AID-1C methodology, helps to maintain direction and momentum in the curricular revision process. For example, at the University of Gondar, this structured methodology allowed the curricular revision process to move forward as planned under the leadership of three different deans, four university administrations, and two national governments. A key benefit of the AID-1C methodology is that it assists in maintaining continuity across different administrations and changes in the VEE.

Another benefit of this methodology is that it was designed to be participatory and collaborative. Throughout the process, the input of external partners is critical to provide a broader and more diverse perspective when evaluating and improving the curriculum. For example, during the assessment (Phase 1), future employers and other key partners from both the public and private sectors are included to help identify gaps and issues that might be missed if only VEE internal participants were included. These external partners are also included in the identification and prioritization of interventions (Phase 3). This helps to ensure that the solutions identified are practical and have the support of the VEE’s external partners. This buy-in is key during the implementation step (Phase 5), where external partners are asked to be part of the solution. This could include providing instruction in a very specialized topic or their area of expertise (e.g., how to prepare official health certificates), mentoring students through field placements, and/or facilitating access to other government or private industry opportunities. This collaborative approach during the curricular revision process also helps to build bridges between students and future employers.

While the Day 1 Competencies serve as a unifying standard for VEEs around the world, it is important to acknowledge that each VEE is unique and will approach the implementation of this standard in different ways. For this reason, the AID-1C methodology provides a general roadmap but is not prescriptive; it is intended to be modified to suit each VEE’s unique circumstances. While advisors from other more experienced VEEs may provide mentorship and consultation regarding the implementation of the AID-1C methodology, the assessment and prioritization are always done by local partners who understand the context in which the VEE’s graduates will be expected to work.

In its current form, the AID-1C methodology is designed as a tool to support focused evaluation of non-clinical competencies. However, it could potentially be adapted to assess other whole-curriculum competency frameworks (e.g., those produced by the Association of American Veterinary Medical Colleges (26) or the European Association of Establishments for Veterinary Education) (27). Since the Assessment phase (Phase 1) of the process was developed specifically to support the evaluation of nonclinical competencies (15), this phase would likely need to be modified to reflect established best practices in the evaluation of clinical competencies [e.g., objective structured clinical evaluation (22–24)].

In conclusion, the AID-1C methodology provides a systematic, collaborative approach through a series of tools and processes that help a VEE evaluate their curriculum, identify, and prioritize gaps, and develop an action plan. This methodology helps VEEs improve their curricula and move toward harmonization with WOAH standards. The result is a curriculum that allows the VEE to train well rounded and competent veterinarians, with the requisite skills to support the veterinary services in their country.

## Data availability statement

The original contributions presented in the study are included in the article, further inquiries can be directed to the corresponding author. Requests to access the datasets should be directed to AH, hoet.1@osu.edu; AMB, berrian.4@osu.edu.

## Author contributions

AH: Conceptualization, Funding acquisition, Methodology, Project administration, Resources, Supervision, Visualization, Writing – original draft, Writing – review & editing. SS: Methodology, Project administration, Writing – original draft, Writing – review & editing. AMB: Conceptualization, Funding acquisition, Methodology, Project administration, Supervision, Writing – original draft, Writing – review & editing. ALB: Methodology, Project administration, Writing – original draft, Writing – review & editing. IG: Project administration, Writing – original draft, Writing – review & editing.

## Footnotes

^1^The term Veterinary Education Establishment (VEE) is used by the World Organization for Animal Health (WOAH) to be more inclusive of different types of institutions that train veterinarians in different countries.
